# Genome-wide association study of resistance to taro leaf blight and yield traits in taro (*Colocasia esculenta* (L.) Schott)

**DOI:** 10.1038/s41598-026-43034-5

**Published:** 2026-03-12

**Authors:** Lydia Jiwuba, Joseph Onyeka, Charles Amadi, Solomon Uko, Abiodun Olutegbe, Simon Peter Abah, Favour Okeakpu, Ezenwanyi Uba, Chiedozie Egesi

**Affiliations:** 1 National Root Crops Research Institute (NRCRI), Umudike, Nigeria; 2 International Institute for Tropical Agriculture (IITA), Ibadan, Nigeria; 3 Department of Plant Breeding and Genetics, Cornell University, Ithaca, NY USA

**Keywords:** Taro (*Colocasia esculenta*), Taro leaf blight (TLB), GWAS, Population structure, Candidate genes, Single nucleotide polymorphisms (SNPs), Tuber yield, Genetics, Plant sciences

## Abstract

Taro leaf blight (TLB), the most destructive disease of taro caused by the oomycete *Phytophthora colocasiae*. An effective strategy for controlling this disease is the development of resistant cultivars. In this study, a genome-wide association study (GWAS) was conducted on 265 taro genotypes to identify genomic regions and putative candidate genes associated with TLB resistance and yield traits. The field experiments were conducted at the National Root Crops Research Institute (NRCRI), Umudike, in 2021/2022 and 2022/2023 planting seasons. TLB severity, plant height, vigor, number of suckers, corm weight, cormel weight, and total tuber weight were evaluated. A genome-wide association mapping detected 18 single nucleotide polymorphisms (SNPs) markers significantly associated with TLB, number of suckers, corm weight, cormel weight, and total tuber weight. Gene annotation of significant SNP loci identified 6, 1, 2, 2, and 1 putative candidate genes associated with TLB resistance, number of suckers, corm weight, cormel weight, and total tuber weight respectively. Co-localization of a significant SNP 83978367 associated with corm weight and total tuber weight on chromosome 11 is possibly an indication of pleiotropic effects or the presence of closely linked genes controlling these traits. GWAS failed to detect significant associations in plant height and vigor. This study provides a basis for research on genetic control of TLB resistance, number of suckers, and yield-associated traits and thus provides resources for the ongoing effort to develop high-yielding, and disease resistant taro cultivars.

**Supplementary Information:**

The online version contains supplementary material available at 10.1038/s41598-026-43034-5.

## Introduction

Taro (*Colocasia esculenta* (L.) Schott), of the family Araceae, is a highly heterozygous, vegetatively propagated crop with a base chromosome number of 14, and commonly found with different ploidy levels in nature, specifically diploid (2n = 28) and triploid (3n = 42)^1^. Diploids are fertile and represent the most common form of taro, while triploids are generally sterile but can be found in various cultivars^2^. Despite being ranked among the most important tuberous crop globally, taro is typically grown on a small scale, and genetic resources are maintained by indigenous smallholder farmers, mainly rural African women with limited resources^3^. Taro is widely cultivated in the tropics and sub-tropics and was first domesticated in Southeast Asia and spread to other parts of the world^4^.

The English term *taro* was borrowed from the Māori (New Zealand) language, where it literally means bread. Taro is an important tuber crop in Nigeria. The corms, cormels, and leaves are edible. Taro corm is an excellent source of carbohydrates, vitamins, proteins, dietary fiber, as well as elements such as potassium, calcium, phosphorus, and iron^5^. Its corm and leaves have some medicinal properties, including those against tuberculosis, ulcers, pulmonary congestion, and fungal infections. It is also consumed by people allergic to cereals and by children who are sensitive to milk. Taro flour is considered a good baby food^5^. Taro is a staple food in many of the Pacific islands, with most of the global production occurring in Nigeria, China, Cameroon, Ghana, and Papua New Guinea, according to FAO statistics 2023^6^. Nigeria accounted for 46% of the 18 million tonnes of taro produced worldwide in 2022. However, Nigeria, the leading producer, faced a reduction from 6.62 tons/ha to 3.91 tons/ha, mainly attributed to repeated outbreaks of diseases such as taro leaf blight (TLB) disease^7^.

The most destructive disease of taro, TLB, is caused by the oomycete *Phytophthora colocasiae*. TLB affects the corms, causing them to rot, and yield losses from this disease can be as high as 70–100%^7^. It also causes significant losses in leaf yield up to 95% in susceptible varieties^8^. In Nigeria, taro is cultivated primarily in the southeastern, southwestern, and northcentral regions with a moist, tropical climate favorable for TLB. Occasional sunlight with intermittent rain is most favorable for disease severity compared to prolonged cloudy weather with rainfall^9^. Several cultural, biological, and chemical measures have been recommended for the control of TLB disease. Each of the measures may have minimal benefits when applied individually, but collectively they can significantly contribute to the integrated approach to disease management. Cultural practices such as pruning the infected leaves during the initial phase of disease progression, wide plant spacing, selecting sites bordered by forest, isolating new crops from the diseased ones, intercropping, and the use of disease-free planting materials^7^. The fungus *Trichoderma* spp. is used as a biocontrol of taro leaf blight disease^10,11^. As for chemical control, a range of systemic fungicides like dithane M-45, copper oxychloride, metalaxyl, and phosphorus acid are the most commonly recommended for the effective control of TLB disease^8^. Fungicides are better applied to the plant on the onset of the disease before it is heavily infected because once the disease enters a symptomatic phase, the potency of disease control is reduced^12,13^. However, the use of fungicides is neither economically sustainable nor environmentally suitable^14^. The use of resistant varieties is the safest and most efficient control measure for TLB in terms of environmental impact and sustainability.

Taro breeding was initiated in many countries of the Pacific, such as Samoa, Papua New Guinea, and Vanuatu, under projects such as TaroGen (Taro Genetic Resources: Conservation and Utilization) and TANSAO (Taro Network for Southeast Asia and Oceania)^15^. Scientists of the National Root Crops Research Institute (NRCRI), Umudike, Nigeria, started taro evaluations as early as 1975 and made some initial attempts at improving local cultivars. Hand pollination is used to produce large numbers of highly heterozygous hybrids that can be selected for disease and desirable agronomic traits. A collection of taro plants maintained by the institute and used in breeding programme includes Nigerian landraces and several cultivars from Samoa and Vanuatu. Over the past few decades, crosses between Nigerian and exotic cultivars have been selected for desirable traits like high yield, taste quality, resistance to pests and diseases, early maturity, flowering ability, and floral productivity, which are used for further breeding^16^. In recent years, taro breeding under the International Network for Edible Aroids (INEA) and the Innovation Lab for Crop Improvement (ILCI) – Feed the Future projects were focused on developing cultivars resistant or tolerant to TLB in NRCRI, Nigeria. One of such crosses (BL158 x NCe004) produced progeny that exhibited TLB resistance. However, conventional phenotype-based modified backcrossing and recurrent selection strategy to breed for TLB resistance is lengthy (it takes up to 10 years or more from pollination to release a new cultivar), labor intensive owing to several biological aspects associated with taro breeding, including poor flowering, low seed set, variation in ploidy and chromosome number, low genetic base and non-uniformity of planting materials. Moreover, the identification of TLB-resistant cultivars through phenotypic selection requires wet environmental conditions that can favor *P. colocasiae* growth. When and where such environmental conditions occur irregularly or non-uniformly, screening for the severity of the disease infection can be difficult. These challenges can be overcome through the use of genomic-assisted and marker-assisted breeding tools to facilitate indirect selection^17^.

There has been limited work on the genetic control of TLB to determine the gene(s) conferring resistance. Most studies on TLB have focused on conventional breeding, with limited work on molecular breeding. Recently, a mapping population ‘1025’ created by crossing two breeding lines ‘230’ and ‘255’, from where ten putative quantitative trait loci (QTLs) were reported to be linked to TLB resistance, was reported^18^. However, the genetic mechanism underlying TLB resistance has not been studied in diversity panels using a genome-wide association mapping approach. Research on TLB resistance using Genome-Wide Association Studies (GWAS) is an emerging area in taro breeding and genetics. GWAS, also known as whole genome association study, is a powerful approach that involves rapidly scanning molecular markers across the complete genomes of different individuals to find genetic variations associated with a specific phenotype^19^. GWAS provides higher mapping resolution than classical bi-parental populations for detecting associations between molecular markers and traits of interest, and has been used to identify alleles that can enhance target traits across a wide range of crops^20^. With the recent development of high-throughput genotyping technologies, genetic variation in many model organisms such as mice, *Arabidopsis*, and maize has been discovered on a genome-wide scale^21^. In modern breeding programs, including those for root and tuber crops, GWAS is a critical tool that aids breeders in developing improved crop varieties faster and more efficiently through marker-assisted selection. For instance, in cassava, GWAS have been conducted on yield components^22^, and it has been used to identify resistance genes to cassava green mite^23^, cassava mosaic disease^17^, and cassava brown streak disease^24^. In their study, Adejumobi et al.^25^ successfully identified genomic regions associated with mosaic virus resistance and tuber yield in *Dioscorea rotundata*. In sweet potato, GWAS have been conducted on yield traits, agronomic traits, weevil resistance, nematode resistance, and anthocyanin content^26–28^. Moreover, Fufa et al.^29^ used a GWAS approach to identify significant SNP loci associated with agronomic traits in taro, including corm diameter, corm length, cormel diameter, cormel length, cormel weight, dry matter, number of cormels per plant, plant height, number of leaves per plant, number of suckers per plant, petiole length and yield.

This study aimed to evaluate the phenotypic responses to TLB and yield-related traits, and identify linked single-nucleotide polymorphisms (SNPs) and putative candidate genes based on a GWAS panel of advanced breeding lines. To the best of our knowledge, this is the first study using GWAS to identify the genomic regions and putative candidate genes associated with TLB resistance in *Colocasia esculenta.*

## Materials and methods

### Plant material, experimental design, and management

The study used a total of 279 taro accessions (Table S1). These accessions represented a diversity panel of taro genotypes developed and collected in the Taro breeding programme of National Root Crops Research Institute, Umudike, Nigeria. They were carefully selected based on their parentage and quantitative traits. Table S1 provides the code, names, and pedigrees of the accessions.

Field experiments were conducted in 2021/2022 and 2022/2023 planting seasons at NRCRI. This experimental site is situated at a latitude 5.4801°N and longitude 7.5437°E; annual rainfall of 2200 mm; mean annual temperature of 22 to 31 °C; Dystric Luvisol soils; humid forest, and altitude of 122 m above sea level. This site represents a hotspot of TLB in the country. The experimental design was a randomized incomplete block design with three replicates. The genotypes were planted at a distance of 1 m × 0.5 m using 10 tubers per genotype. Each plot consists of two rows, each 5 m long. The row spacing was 1 m apart, and spacing within the rows was 0.5 m to maximize sucker production, which serves as planting material.

NPK 15:15:15 fertilizer was applied at 600 kg per hectare at 6 weeks after planting by the ring method. During the season, the field was manually weeded three times.

### Phenotypic evaluation

Phenotypic variables such as TLB severity, corm weight, cormel weight, total tuber weight, number of suckers, plant height, and vigor were measured under natural field conditions at Umudike which is a hotspot for TLB disease. Data were recorded at multiple growth stages using standardized protocols as shown in Table 1.

**Table 1:** Description of the studied traits.

Trait type	Trait name	Abbreviation	Description of trait
Stress trait (categorical)	Taro leaf blight severity	TLBS	Visual severity rating of plants infected with TLB caused by oomycete *Phytophthora colocasiae*. Symptoms rated from 1 = clean, no infection. 2 = Up to 25% leaf area infected, mild leaf necrosis. 3 = 25 – 50% leaf area infected, moderate leaf necrosis. 4 = 50 – 70% leaf area infected, severe leaf necrosis, severe coalesced spots. 5 = 75 – 100% leaf area infected, severe coalesced spots, collapse of petiole, complete leaf blight. TLBS was evaluated at 2, 4 and 6 months after planting (MAP)
Agronomic trait (categorical)	Vigor	Vig	Visual rating at the scale of 3,5 and 7 with 3 = not vigorous, 5 = Vigorous and 7 = highly vigorous. Vigor was evaluated at 2, 4 and 6 MAP
Agronomic trait (continuous)	Plant height	Plt ht	Measured in cm as height from ground level to the top of canopy and evaluated at 2, 4 and 6 MAP
Agronomic trait (continuous)	Number of suckers	Num_suckers	Counting the number of suckers per plot at harvest (8 MAP)
Agronomic trait (continuous)	Corm weight	Corm_wt	Weight of corm from a unit plot (scale g) at harvest (8 MAP)
Agronomic trait (continuous)	Cormel weight	Cormel_wt	Weight of cormel from a unit plot (scale g) at harvest (8 MAP)
Agronomic trait (continuous)	Total tuber weight	Total tuber wt	Weight of tuber (corms and cormels) from a unit plot (scale g) at harvest (8 MAP)

### DNA extraction and SNP genotyping

#### Sample collection and shipping

Before sampling, 279 healthy plants were tagged with barcode labels for proper identification. For each sample, 4 leaf disks of 6 mm diameter punches were collected from the healthy young green tissue using the paper puncher and placed per well in a 96-well labeled plate on wet ice. After punching each leaf sample, the paper puncher was wiped with a paper towel dampened with 70% ethanol to avoid cross-contamination during sample collection. The samples were taken to the NRCRI Biosciences molecular laboratory for lyophilization for 24–48 h, as this results in the best quality DNA. The plates were sealed with silicone seals, wrapped in plastic bags, and secured with rubber bands. Silica packs were added to the plastic bags to keep the samples dry during shipment to Intertek AgriTech Laboratory in Australia for DNA extraction.

The DNA extraction was performed by Intertek Agritech using sbeadex chemistry by Biosearch Technologies as per the manufacturer’s instructions (www.intertek.com). The extracted genomic DNA samples were sent to Diversity Arrays Technology (DArT) Pty Limited in Canberra, Australia (www.diversityarrays.com) for DArTSeq genotyping.

#### SNP filtering

Raw SNP data generated by DArT were subjected to a stepwise marker screening and quality control procedure prior to analysis. Loci and individual were filtered using the dartR package^30^ in R version 3.6.1^31^, R Core Team, 2020). To eliminate low-quality SNPs, markers with a missing call rate greater than 20% were removed. Monomorphic SNPs (loci with only one allele), which do not provide useful genetic information, were removed, and SNPs with minor allele frequency of 5% were also removed to minimize the influence of rare alleles and potential genotyping errors. Sequence read depths was further screened using DArT default settings, and SNPs with read depths below 5 or above 50 were excluded, as low or excessively high read counts can lead to unreliable genotype^32^. SNPs with an average reproducibility of technical replicate assay pairs that fell below 99% were also removed. To reduce redundancy arising from highly similar sequences, SNPs with pairwise Hamming distance of 0.2 were removed^33^. Finally, markers that did not conform to the expectations of Hardy–Weinberg Equilibrium (HWE) were removed. The resulting filtered SNP dataset was used for population structure analysis, linkage disequilibrium, and genome-wide association analysis.

### Data analysis

## Descriptive statistics

In preparing the dataset for analysis, data wrangling was done using the tidyverse^34^ package in R (R Core Team 2023). Descriptive statistics, including mean, range, standard deviation (SD), and coefficient of variation (CV), were evaluated for each trait across genotypes over two planting seasons. These analyses were done using the dplyr (v1.1.4;^35^) and tidyr (v1.3.1;^35^) packages. Phenotypic distributions were visualized using boxplots to assess trait variability, identify outliers, and compare distributions between years prior to genome-wide association analysis.

### Trait correlation

Pearson’s correlation coefficients were calculated using R, employing the psych package^36^ to evaluate the relationships among measured traits while chart. Correlation function in the Performance Analytics package^37^ was used to visualize the correlation matrix.

### Principal component analysis and trait biplot

To assess relationships among the traits and explore genotype variation based on multivariate performance, principal component analysis (PCA) was conducted using prcomp () function in R version 4.3.1, with the argument scale. = TRUE to ensure that all traits contributed equally regardless of their original scales. The resulting principal components were used to visualize the multivariate structure of the data and identify traits contributing most to variation among genotypes.

A PCA biplot was generated using the fviz_pca_biplot () function from the *factoextra* package in R. In the biplot, genotypes are represented as points and traits as vectors (arrows), which indicate both the direction and strength of each trait’s contribution to the first two principal components. Genotypes positioned close to a trait vector suggest a strong expression for that trait, while the angle between traits vectors illustrates their suggest correlation among traits. This multivariate approach enabled a comprehensive evaluation of trait interrelationships and clustering patterns among genotypes based on phenotypic performance.

### Population structure and genetic diversity analysis

Population structure was done using the model-based clustering algorithm implemented in ADMIXTURE version 1.3.0^38^. The filtered SNP dataset was first converted into PLINK format using PLINK v1.9. ADMIXTURE was run with K values (number of ancestral populations) ranging from 2 to 5, and each run replicated to ensure consistency. The optimal number of clusters (K) was determined by selecting the value with the lowest cross-validation (CV) error as the best -fit model. The estimated proportions for each individual were generated as bar plots in R. To further assess hierarchical population structure, the Evanno ΔK method was applied to identify the strongest upper-level genetic subdivision by estimating ΔK based on the rate of change in model likelihoods across successive K values.

To complement the model-based clustering results, a genomic relationship matrix was constructed using the *ASRgenomic* package (version 1.0.0) in R, based on the VanRaden method. Additionally, a genetic distance matrix was generated using the dist() function in R. A dendrogram for hierarchical clustering was constructed using the Ward’s minimum variance method (hclust, method = “ward.D2”) in R to visualize genetic relationships among genotypes.

Genetic differentiation (Fst) among inferred clusters was estimated using analysis of molecular variance (AMOVA) in GenAlEx 6.053 with 999 permutations.

Genetic diversity among the taro genotypes was quantified using metrics such as expected heterozygosity (He), observed heterozygosity (Ho), and polymorphic information content (PIC), which were calculated using the Tassel 5.0 software. The combined approaches provided a comprehensive view of the genetic structure and diversity present in the taro panel, supporting further genome-wide association (GWAS) and breeding applications.

### Linkage disequilibrium analysis

Linkage disequilibrium (LD) was estimated in TASSEL 5.0 by calculating pairwise r^2^ values between SNPs. The output, which included r^2^ and the physical distance between marker pairs, was exported and used to produce the LD decay plot in R. In the decay plot, r^2^ was plotted against physical distance, and the point at which r^2^ dropped below 0.20 was taken as the LD decay distance.

#### Genome-wide association studies

Genome-wide association studies were conducted using the GAPIT (Genome Association and Prediction Integrated Tool) package in R to identify significant marker-trait associations for the traits observed in this study. The panel comprised 265 taro accessions evaluated at Umudike across two years, and 7395 SNP markers, which had been filtered to retain markers with an MAF greater than or equal to 0.05 and a missing call rate greater than or equal to 20%.

To account for population structure and kinship, a mixed linear model (MLM) was fitted in GAPIT. The model incorporated both the population structure matrix (Q) derived from principal component analysis (PCA) and the kinship matrix (k), estimated using the VanRanden method. The model equation was:

$$y = X\beta + Zu + e$$

where y is the vector of phenotypic values.

X is the fixed effects (SNP markers and population structure covariates).

β is the vector of fixed-effect coefficients.

Z is the design matrix for random genetic background effects.

u is the vector of random additive genetic effects.

ε is the random error.

The random effects were assumed to follow a normal distribution: u∼N (0, Kσ^2^u) and ε ~ Ν (0, Ισ^2^ε).

Significance thresholds were adjusted using false discovery rate (FDR) control to reduce the likelihood of false positives. Manhattan and quantile–quantile (QQ) plots were generated to visualize the genomic locations of significant SNPs and deviations of observed *p*-values from the expected null distribution. For each significant SNP, the phenotypic variance explained (PVE) was estimated using the coefficient of determination (R^2^) from a linear model.

Broad-sense heritability (H^2^) was estimated for each trait from a combined analysis across two years using the formula below, which accounts for genetic, environmental, and interaction variances on a plot basis.

$${H}^{2}=\frac{{\sigma }^{2}g}{{\sigma }^{2}g+\frac{{\sigma }^{2}gy}{ny}+\frac{{\sigma }^{2}e}{nr.ny}}$$

where *σ*^2^*g* is the genetic variance, *σ*^2^*gy* is the variance due to the genotype by year interaction, *ny* is the number of years, *nr* is the number of replications, and *σ*^2^*e* is the residual variance.

#### Candidate gene identification and functional annotation

The significant SNPs were scanned for potential candidate genes underlying the studied traits based on the gene ontologies and predicted functions. Gene ontology annotation was done using the National Center for Biotechnology Information (NCBI) SNP database (dbSNP). The SNP identifiers (IDs) corresponding to significant GWAS results were individually queried using the NCBI db SNP search engine (https://www.ncbi.nlm.nih.gov/snp). Genes located within a ± 6 Mb window were considered putative candidates, corresponding to the estimated LD decay distance. Gene descriptions, predicted protein functions, and biological processes were extracted from the NCBI Gene database. Orthologous genes from related species were also examined to support functional predictions.

## Results

### Phenotypic variation and heritability of traits

**Fig. 1:**
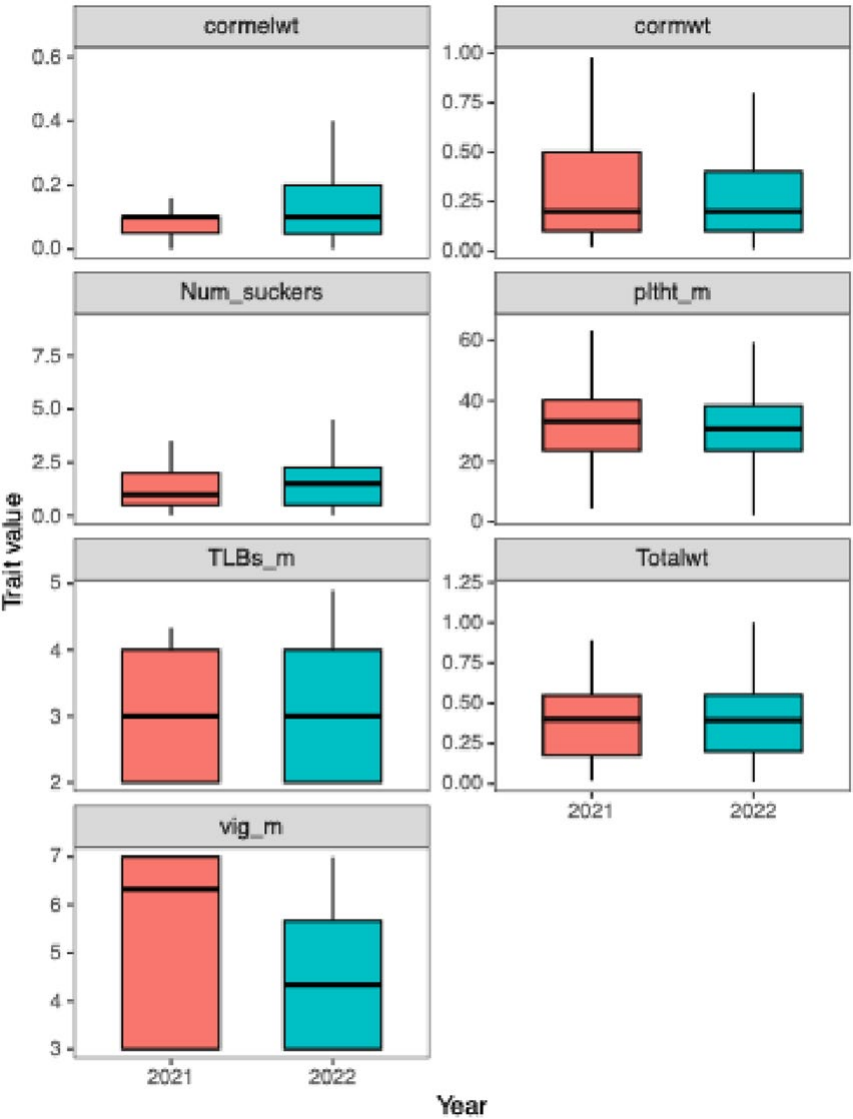
The distribution of cormel weight (cormelwt), number of suckers (Num_suckers), taro leaf blight severity mean (TLBs_m), vigor mean (vig_m), corm weight (cormwt), plant height mean (pltht_m) and Total weight (Totalwt).

A wide range of phenotypic variation was observed among the taro genotypes for all the traits evaluated (Fig. 1, Table 2). The coefficient of variation (CV) across traits ranged from 18.52% for taro leaf blight severity at 6 months after planting (TLBs6MAP) to 110.5% for cormel weight, indicating considerable variability within the population.

**Table 2:** Descriptive statistics (mean, minimum (min), maximum (max), standard deviation (SD), coefficient of variation (CV)) and broad sense heritability (H^2^) estimate for evaluated traits across two years in Umudike.

Trait	Mean	Min	Max	SD	CV	H^2^
TLBs2MAP	2.64	1.00	5.00	0.97	36.81	6
TLBs4MAP	2.59	1.00	5.00	1.09	42.17	14
TLBs6MAP	3.32	3.00	5.00	0.62	18.52	4
TLBs_mean	2.85	1.56	5.00	0.56	19.51	21
Vig2MAP	4.49	3.00	7.00	1.49	33.07	26
Vig4MAP	5.70	3.00	7.00	1.53	26.87	30
Vig6MAP	5.27	3.00	7.00	1.62	30.67	38
Vigor mean	5.15	3.00	7.00	1.25	24.30	66
Pltht2MAP	30.49	9.00	57.59	10.43	34.21	71
Pltht4MAP	58.55	9.52	111.31	17.97	30.68	98
Pltht6MAP	47.78	14.00	65.00	11.92	24.95	54
Pltht mean	40.27	4.37	65.82	12.89	32.00	80
Num_suckers	1.97	0.16	3.99	0.98	49.62	21
Cormel_wt	0.31	0.01	1.50	0.34	110.50	22
Corm_wt	0.34	0.01	2.50	0.32	95.52	14
Total_ wt	0.58	0.01	4.00	0.54	92.00	24

TLBs2MAP: Taro leaf blight severity at 2 months after planting; TLBs4MAP: Taro leaf blight severity at 4 months after planting; TLBs6MAP: Taro leaf blight severity at 6 months after planting; TLBS_mean: mean severity of taro leaf blight; Vig2MAP: vigor at 2 months after planting; Vig4MAP: vigor at 4 months after planting; Vig6MAP: vigor at 6 months after planting; vigor mean: mean value of vigor; Pltht2MAP: plant height at 2 months after planting; Pltht4MAP: plant height at 4 months after planting; Pltht6MAP: plant height at 6 months after planting; Pltht mean: mean value of plant height; Num_suckers: number of suckers; Cormel_wt: Cormel weight; Corm_wt: corm weight; Total_wt: total tuber weight.

Taro leaf blight severity (TLBs) measured on a scale of 1–5 showed moderate to high variation, with mean scores increasing over time from 2 months after planting (mean = 2.64) to 6 months after planting (mean = 3.32). The overall mean TLB score (TLBs_mean) was 2.85, indicating moderate disease pressure under natural field infestation. The heritability estimates for these traits were relatively low, ranging from 4 to 21%, suggesting strong environmental influence and weak genetic control.

Vigor at 2, 4, 6 MAP, and the mean of vigor showed moderate variability (CV = 24–33%), with broad-sense heritability estimates ranging from 26% (Vig2MAP) to 66% (vigor mean). This indicates a good level of genetic control and potential for selection.

Plant height traits such as plant height at 2 MAP (Pltht2MAP), plant height at 4 MAP (Pltht4MAP), plant height at 6 MAP (Pltht6MAP), and mean value of plant height (Pltht mean) exhibited high genetic variation, with heritability estimates ranging from 54 to 98% and a CV of 25–34%. The highest heritability (H^2^ = 98%) was observed at Pltht4MAP, suggesting strong and consistent genetic expression at this stage.

Yield components such as cormel weight (cormel_wt), corm weight (corm_wt), and total tuber weight (total tuber_wt) showed very high variability with CVs above 90%. The number of suckers exhibited a lower CV (49.62%) than the yield traits. Heritability estimates for the yield traits were low to moderate, ranging from 14% (corm_wt) to 24% (total tuber_wt).

Overall, the performance of plants that were moderately resistant to TLB was greater than the number of plants that were either resistant or susceptible.

### Principal component analysis and trait relationships

**Fig. 2:**
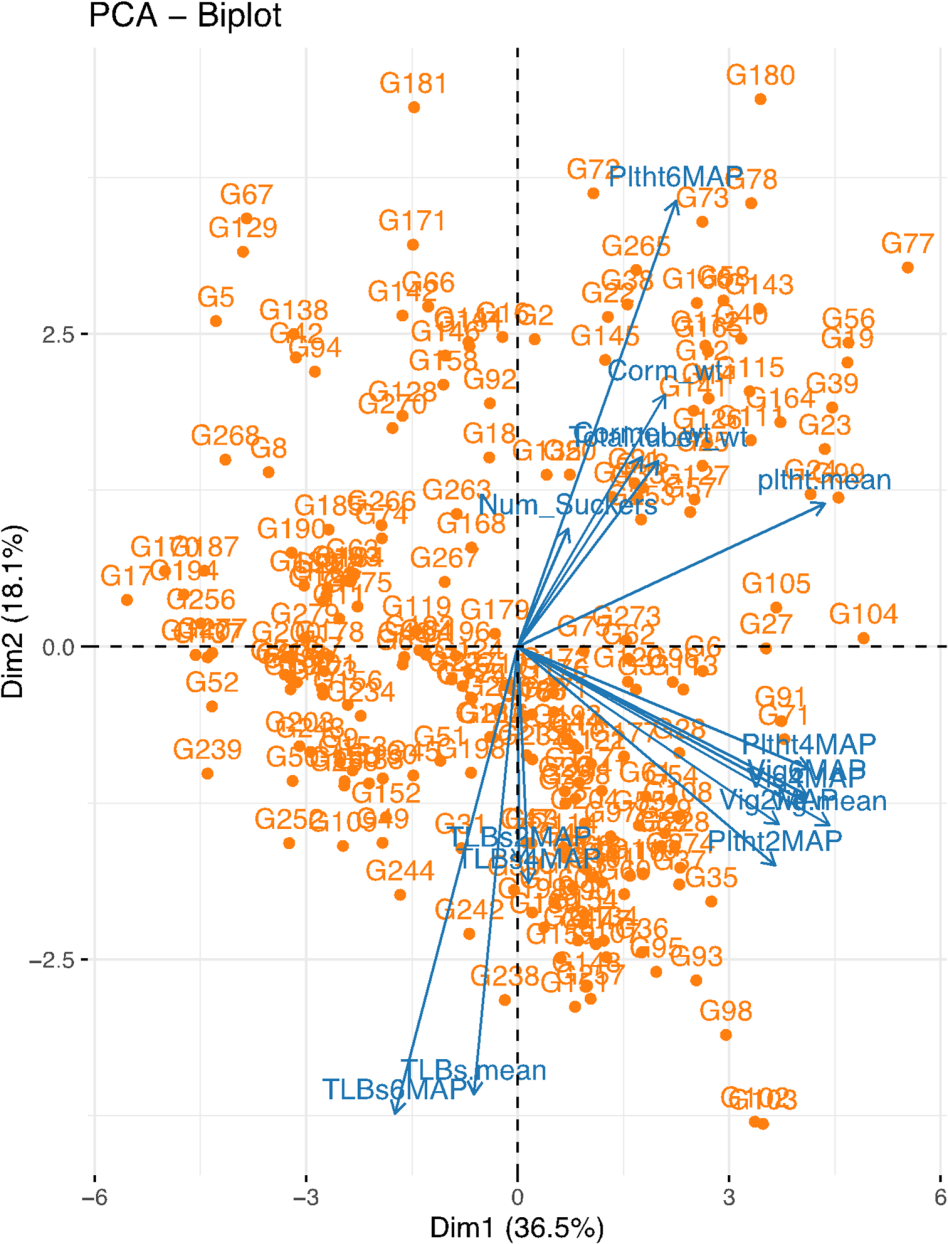
Trait PCA-Biplot. TLBs2MAP, Taro leaf blight severity at 2 months after planting; TLBs4MAP, Taro leaf blight severity at 4 months after planting; TLBs6MAP, Taro leaf blight severity at 6 months after planting; TLBS_mean, mean severity of taro leaf blight; Vig2MAP, vigor at 2 months after planting; Vig4MAP, vigor at 4 months after planting; Vig6MAP, vigor at 6 months after planting; vigor mean, mean value of vigor; Pltht2MAP, plant height at 2 months after planting; Pltht4MAP, plant height at 4 months after planting; Pltht6MAP, plant height at 6 months after planting; Pltht mean, mean value of plant height; Num_suckers, number of suckers; Cormel_wt, Cormel weight; Corm_wt, corm weight; Total tuber_wt, total tuber weight. The name of the genotypes is indicated as G1, G2, G3, ……, G279 (Table S1).

To assess phenotypic variability and trait associations across taro genotypes, a principal component analysis (PCA) was conducted. The first two principal components (PC1 and PC2) accounted for 36.6% and 18.1% of the total variation, respectively, cumulatively explaining 54.7% of the phenotypic variation in the dataset (Fig. 2).

The PCA biplot revealed distinct patterns of trait interrelationships and phenotypic diversity among the genotypes. Yield–related traits, including corm weight, cormel weight, and total tuber weight, showed strong position loadings along PC1, indicating that they contributed substantially to the observed phenotypic variation and were positively correlated. Genotypes with the codes G4, G253, G33, G141, G126, G25, G127, G57 and G12 had high values for the yield traits.

Vigor and plant height traits measured at different growth stages were dispersed across both PC1 and PC2 and clustered closely with the yield traits. This suggests a potential positive contribution of vigor and plant height to yield performance. Genotypes G180, G98, G91, G71, G28, G24, G99, G73, G172, G271, G145, G265, G38, G78, G73 and G22 performed best for growth parameters.

Taro leaf blight severity evaluated at 2, 4, and 6 MAP, projected in the opposite direction to the yield traits, indicating a negative correlation between disease severity and yield. Genotypes located in that quadrant were more susceptible and potentially lower yielding.

### Trait correlation analysis

**Fig. 3:**
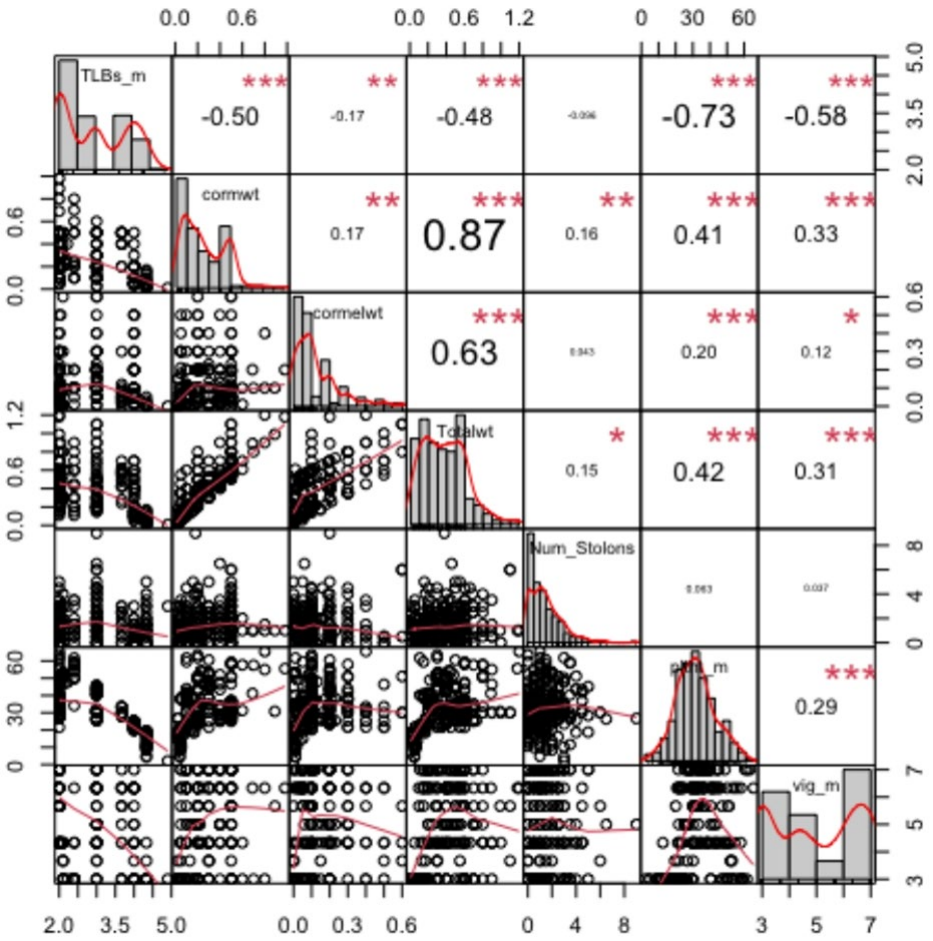
Pearson correlation matrix showing the relationships among taro leaf blight severity and other agronomic traits. TLBs_m, Taro leaf blight severity mean; cormwt, corm weight; cormelwt, cormel weight; totalwt, total tuber weight; Num_suckers, number of suckers; pltht_m, plant height mean; vig_m, vigor mean. ***significant at *P* < 0.001, **significant at *P* < 0.01, *significant at *P* < 0.05, ns, not significant.

Pearson’s correlation analysis showed significant relationships among the phenotypic traits evaluated across the taro genotypes (Fig. 3). Total tuber weight showed significant and positive correlations with cormel weight (r = 0.63, *p* < 0.001), corm weight (r = 0.87, *p* < 0.001), and number of suckers (r = 0.15, *p* < 0.05), indicating that these traits substantially contribute to the tuber yield performance. In addition, total tuber weight was also positively associated with the mean value of plant height (r = 0.42, *p* < 0.001) and the mean value of vigor (r = 0.31, *p* < 0.001). A significant positive correlation was observed between vig_m and plt ht_m (r = 0.29, *p* < 0.001). Conversely, the results showed that corm_wt (r = − 0.5), cormel_wt (r = − 0.17), total_wt (r = − 0.48), plt ht_m (r = − 0.73) and vig_m (r = − 0.58) significantly and negatively correlated with TLBs_m (*p* < 0.001). The results also showed that plants with severe TLBs had reduced vigor and lower tuber yield.

### Genotypic analyses

#### SNPs summary

The initial number of loci and individuals provided by DArT Pty was 25532 and 279, respectively. After processing and filtering, 7395 loci and 265 individuals were used to assess the genetic diversity among the taro genotypes. Table 3 showed that the minor allele frequency (MAF) varied from 0.0019 to 0.4754, with an average of 0.0575. The expected heterozygosity (He) ranged from 0.0192 to 0.2964, with a mean of 0.11283, while the observed heterozygosity (Ho) ranged from 0.0032 to 0.5623 (mean of 0.1128). The polymorphic information content (PIC) values ranging from 0 to 0.500 have a mean of 0.0102.

**Table 3:** SNP marker descriptive statistics (minor allele frequency (MAF), expected heterozygosity (He), observed heterozygosity (Ho), polymorphic information content (PIC)).

Descriptive Statistics	MAF	He	Ho	PIC
Minimum	0.0019	0.0192	0.0032	0.0000
Maximum	0.4754	0.2946	0.5623	0.5000
Mean	0.0574	0.1128	0.1128	0.0102

### Population structure and genetic diversity

**Fig. 4:**
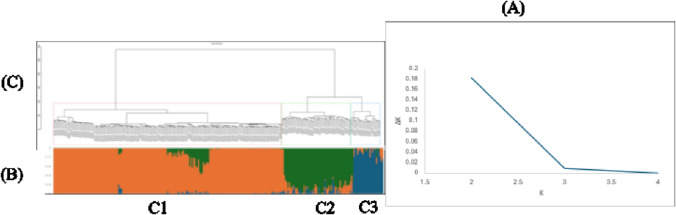
Population structure and genetic analysis showing (**A**) distribution of delta k values across different K values; (**B**) the admixture plot for k = 3; and (**C**) hierarchical clustering dendrogram of individuals divided into three main clusters at h = 100.

ADMIXTURE results were evaluated using cross-validation error (Fig. 4B) and an ADMIXTURE-based Evanno approximation for the population structure analysis (Fig. 4A). Evaluation of model predictive accuracy using cross-validation (CV) error indicated that K = 3 provided the best fit to the data, as evidenced by the lowest CV error among the tested values of K while the Evanno method showed a pronounced peak in ΔK at K = 2, indicating the strongest upper-level genetic division within the panel corresponds to two major lineages (Fig. 4A). This represents the first hierarchical level of structure.

However, increasing K from 2 to 3 revealed additional, biologically meaningful substructure within one of the major lineages identified at K = 2. The K = 3 grouping provided clearer separation of genotypes and aligned more closely with known geographic, ecological, and breeding histories, supporting its biological relevance. Such a pattern is indicative of a hierarchical population structure in which two major genetic groups are further subdivided into finer subpopulations. The retention of K = 3 was therefore justified not only by statistical support from cross-validation but also by its ability to reduce confounding due to cryptic relatedness and population stratification. Accounting for this finer-scale structure is particularly important in association mapping studies, to avoid false-positive associations.

Based on the combined evidence from ADMIXTURE cross-validation (CV) error, the Evanno ΔK method, and biological interpretability, K = 3 was selected as the most appropriate number of clusters. This classification divided the taro genotypes into three major genetic groups (C1, C2, and C3), as illustrated in Fig. 4B and C. Clusters 1, 2, and 3 comprised 69.81%, 21.13%, and 9.06% of the total genotypes, respectively (Table S2). To validate the population structure, a hierarchical clustering dendrogram (cut at h = 100) was also generated. Each cluster was outlined in different colors. Red color represents cluster 1, green color represents cluster 2, while blue color represents cluster 3 (Fig. 4C). These clusters represent groups of genotypes that share genetic similarity, with shorter branch lengths indicating a higher degree of relatedness among individuals within the same cluster.

The dendrogram confirmed that cluster 1 was the largest, with 185 genotypes (Fig. 4C, Table S2). Most genotypes assigned to cluster 1 are descendants from a mixture of Vanuatu–Nigerian and Vanuatu-Samoa backgrounds. Cluster 2 contained 56 genotypes with ancestry tracing mainly to Vanuatu and Samoa. Cluster 3 formed a distinct subpopulation consisting of 24 genotypes, which are derived from only Samoa ancestry (Fig. 4C, Table S2).

The three clusters obtained from the structural analysis were subsequently exposed to AMOVA in order to determine the variation among populations, among individuals and within individuals. While a 1%variance was observed among populations, 59% variance was observed among individuals and 40% within individuals was observed (Table 4). Genetic differentiation (Fst) among the three inferred clusters was low but significant, with an overall Fst of 0.013 (*P* = 0.003) (Table 4). This level of differentiation is consistent with the ADMIXTURE results and indicates only weak population subdivision within the panel, supporting its suitability for GWAS.

**Table 4:** Analysis of molecular variance (AMOVA) summarizing the distribution of genetic variation among populations, among individuals within populations, and within individuals in the taro diversity panel based on genome-wide SNP data. Associated F-statistics and significance levels are also shown.

Source	df	SS	MS	Est. Var	%	F-Statistics	Value	*P* (rand > = data)
Among Pops	2	2036.788	1018.394	4.155	1%	Fst	0.013	0.003
Among Indiv	262	134172.372	512.108	191.374	59%	Fis	0.597	0.001
Within Indiv	265	34280.500	129.360	129.360	40%	Fit	0.602	0.001
Total	529	170489.660		324.890	100%			

df: degree of freedom; SS: sum of squares; MS: mean squares; Est.Var.: estimate of variation; Fst: fixation index/genetic differentiation; Fis: inbreeding coefficient within subpopulations; Fit: total inbreeding coefficient.

### Linkage disequilibrium decay

**Fig. 5:**
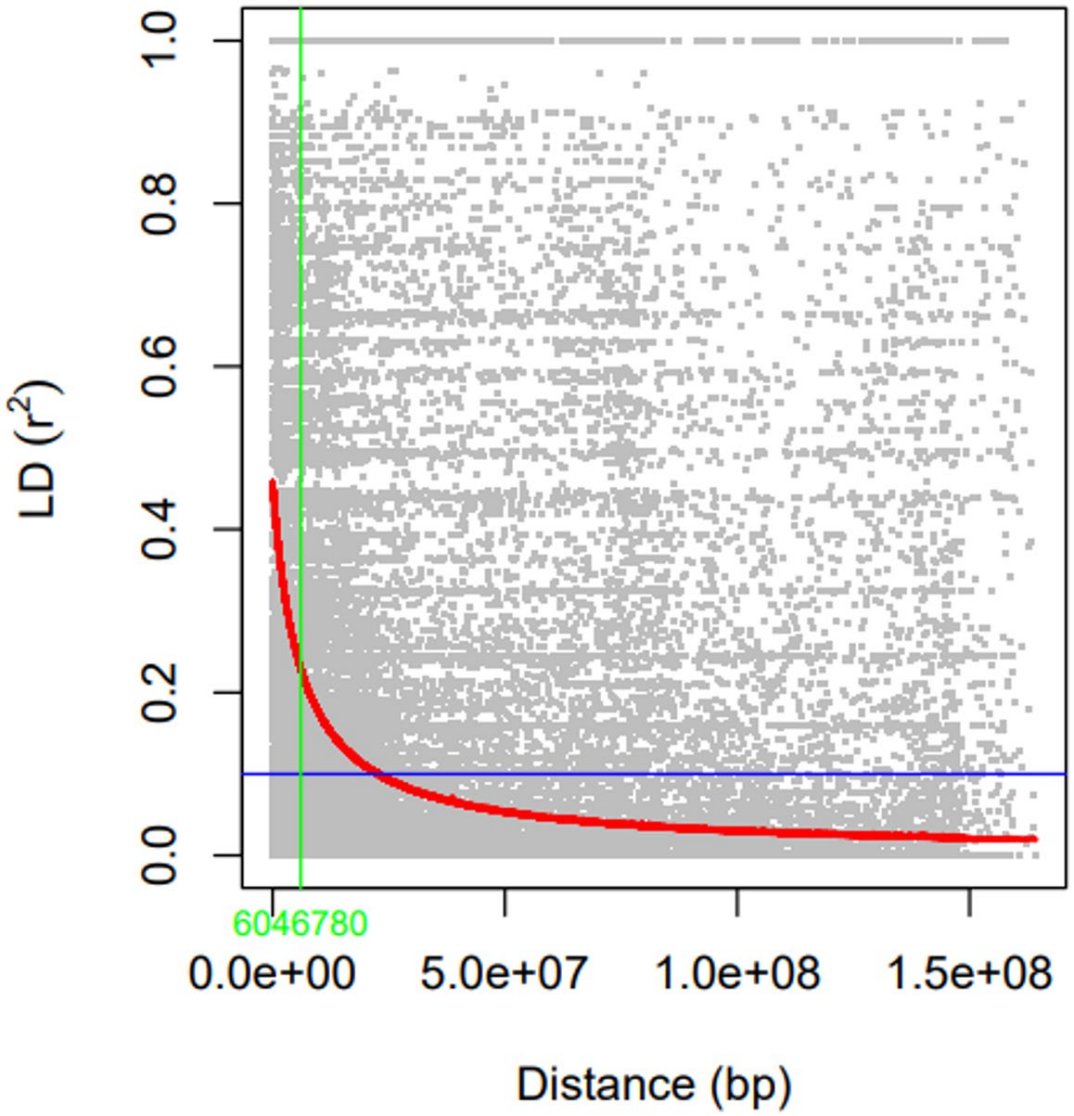
Linkage disequilibrium (LD) decay in the taro association panel. Pairwise LD (r^2^) is plotted against physical distance (bp) between SNPs. The red line represents the locally weighted regression (LOESS) curve, the horizontal blue line indicates the background LD threshold and the vertical dashed line marks the estimated LD decay distance.

Linkage disequilibrium (LD) decay was evaluated to assess the genomic resolution of the GWAS panel (Fig. 5). Pairwise LD, measured as the squared correlation coefficient (r^2^), declined with increasing physical distance between SNPs. The average r^2^ dropped below 0.20 at around 6 Mb, after which LD approached background levels (Fig. 5). This pattern indicates relatively fast LD decay in the panel and supports the mapping resolution used for the GWAS.

### Genome-wide association study (GWAS)

**Fig. 6:**
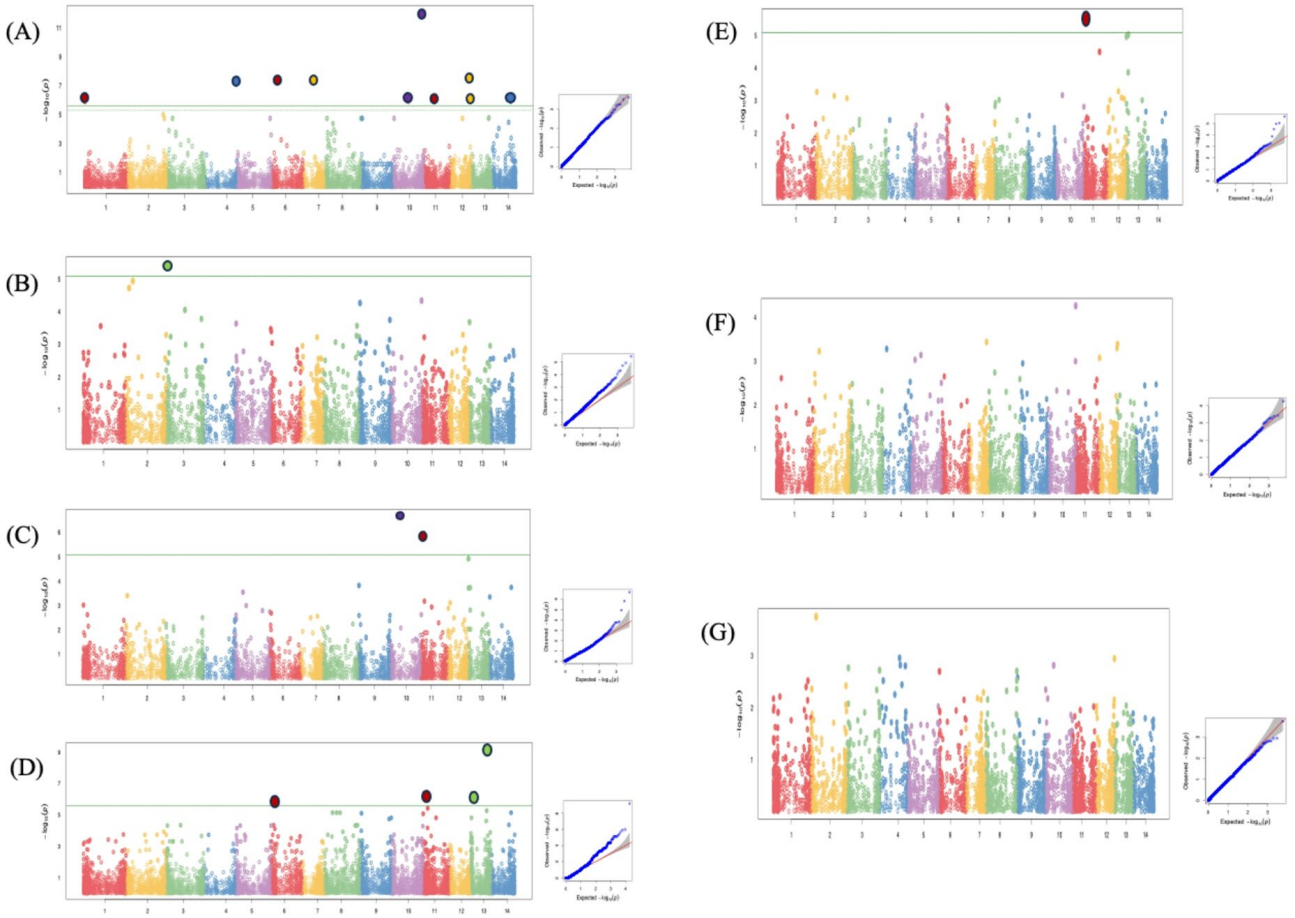
Manhattan plots summarizing genome-wide association results for (**A**) taro leaf severity, (**B**) number of suckers, (**C**) corm weight, (**D**) cormel weight, (**E**) total tuber weight, (**F**) plant height, and (**G**) vigor. Horizontal line on the plot indicates significance threshold. The X-axis is the genomic position of the SNPs in the genome, and the Y-axis is the negative log base 10 of the *P*-values. Each chromosome is colored differently. SNPs with stronger associations with the trait will have a larger Y-coordinate value.

GWAS analysis results for all the phenotypic traits evaluated across years are presented using the Manhattan plots (Fig. 6) which illustrates the distribution of -log_10_ (*p*-values) for each single nucleotide polymorphism (SNP) across all chromosomes. Each point represents an individual marker, with alternating colors used to differentiate chromosomes for visual clarity.

### Taro leaf blight severity (TLBS)

A total of ten loci were significantly associated with resistance to taro leaf blight (TLB). One significant SNP was identified on each of chromosomes 1, 4, 6, 7, 11, and 14, while two SNPs were detected on chromosomes 10 and 12. The strongest association was observed on chromosome 10, where SNP 18912932 showed the most significant signal with a *p*-value of 8.86 × 10^−13^ (Fig. 6A, Table 5).

**Table 5:** GWAS results for all significant SNPs.

Traits	SNP	Chr	SNP Position	*P*.value	MAF	Effect
TLB	18912932|F|0–21:A > G	10	136908522	8.86E-13	0.496	− 0.121
TLB	100098210|F|0–10:A > C	4	151847109	4.28E-08	0.498	− 0.121
TLB	18914010|F|0–14:G > A	6	24061444	4.28E-08	0.498	− 0.121
TLB	100162979|F|0–17:G > C	7	58127735	4.28E-08	0.498	− 0.121
TLB	77764140|F|0–67:G > A	12	92391495	4.28E-08	0.498	− 0.121
TLB	100025035|F|0–39:T > C	1	2653855	1.26E-06	0.498	− 0.120
TLB	100014530|F|0–43:G > A	10	76334727	1.26E-06	0.498	− 0.120
TLB	100019468|F|0–45:A > G	11	52506771	1.26E-06	0.498	− 0.120
TLB	83984858|F|0–8:T > A	12	101003695	1.26E-06	0.498	− 0.120
TLB	83979190|F|0–16:T > G	14	91199766	1.26E-06	0.498	− 0.120
Num_suckers	100014346|F|0–58:T/C	3	11597984	3.58E-06	0.004	4.397
Corm weight	100026239|F|0–31:C/T	10	36200803	2.15E-07	0.004	0.529
Corm weight	83978367|F|0–64:T/C	11	6601002	1.57E-06	0.006	0.406
Cormel weight	100028428|F|0–41:T > C	13	91641212	6.14E-10	0.488	− 0.331
Cormel weight	83979002|F|0–12:T > C	11	26472718	1.02E-06	0.486	− 0.220
Cormel weight	18911820|F|0–20:T > C	13	5933613	1.22E-06	0.493	− 0.323
Cormel weight	100202114|F|0–52:C > A	6	17055530	2.05E-06	0.493	− 0.308
Total tuber wt	83978367|F|0–64:T/C	11	6601002	2.59E-06	0.006	0.563

### Number of suckers

We found a single SNP marker on chromosome 3 to be associated with the number of suckers and is tagged by SNP 100014346 (*p* value of 3.58 × 10^−6^) (Fig. 6B, Table 5).

#### Corm weight

Two SNPs displayed significant associations with corm weight (Fig. 6C). These SNPs (100026239 and 83978367) were found on chromosomes 10 and 11 with *p* values of 2.15 × 10^−7^ and 1.57 × 10^−6^, respectively (Table 5).

#### Cormel weight

GWAS for cormel weight uncovered four association signals (Fig. 6D). Two SNPs were located on chromosome 13 (SNP 100028428, *p* value of 6.14 × 10^−10^ and SNP 18911820, *p* value of 1.22 × 10^−6^). The remaining SNPs were located on chromosome 11 (SNP 83979002, *p* value of 1.02 × 10^−6^) and chromosome 6 (SNP 100202114, *p* value of 2.05 × 10^−6^) (Table 5).

#### Total tuber weight

A SNP marker was found to be significantly associated with the total tuber weight. This SNP 83978367 with a *p* value of 2.59 × 10^−6^ was found on chromosome 11 (Fig. 6E, Table 5). However, the same SNP 83978367 was also significantly associated with another trait (corm weight), suggesting the presence of pleiotropic loci or tightly linked genomic regions. Thus, chromosome 11 had associations for total tuber weight and corm weight.

In total, 18 SNPs were significantly associated with the traits evaluated. The GWAS identified 10, 1, 2, 4, and 1 SNP markers for TLB resistance, number of suckers, corm weight, cormel weight, and total tuber weight, respectively. The most significant association was detected for SNP 18912932, with a -log_10_ (*p* value) of 8.86 × 10^−13^. No significant SNPs were observed for vigor and plant height in this population.

A quantile–quantile plot of the *p*-values is shown to assess the number and magnitude of observed associations between genotyped single-nucleotide polymorphisms and the traits.

#### Putative candidate gene identification and functional annotation

The most significant SNPs were further investigated for the presence of potential candidate genes using NCBI SNP database to retrieve gene annotations. Eleven unique genes were identified with SNPs associated with TLB resistance, number of suckers, corm weight, cormel weight and total tuber weight. Among these genes, six candidate genes showed association with taro leaf blight resistance. In Table 6, the six candidate genes were located on chromosome 10 harboring the Ferredoxin reductase (FDXR); on chromosome 4, harboring the Dihydrolipoamide dehydrogenase (DLD); on chromosome 12, harboring Orinithine decarboxylase antizyme 2 (Oaz2); on chromosome 14, harboring Transcient receptor potential (TRP-like); on chromosome 7, harboring cathepsin B-like cysteine proteinase 5; and on chromosome 10 harboring Ras oncogene family (Rab7b gene).

**Table 6:** Genes annotated as candidates for association with TLB resistance, number of suckers, corm, cormel and total tuber weights.

Trait	SNP marker	Chr	Gene	Gene function
TLB	SNP 18912932	10	FDXR; Ferredoxin reductase	Photoprotection and stress resilience
	SNP 100098210	4	DLD; Dihydrolipoamide dehydrogenase	A key enzyme in cellular energy metabolism, ensuring the efficient breakdown of various molecules and the subsequent production of ATP
	SNP 83984858	12	oaz2; ornithine decarboxylase antizyme 2	They are polyamine regulator which are crucial for plant’s ability to adapt to and survive under stress conditions
	SNP 83979190	14	TRP—like; transient receptor potential	Cellular sensors, contributes to plant responses to stress, nutrient uptake and cell signaling
	SNP 100162979	7	Cathepsin B—like cysteine proteinase 5	Plays a vital role in hypersensitive response and apoptosis
	SNP 100014530	10	Rab7b, Ras oncogene family	Involved in biotic and abiotic stress resilience, as well as developmental regulation
Number of suckers	SNP 100014346	3	eIF—2—alpha kinase activator GCN1; General Control Non-derepressible 1	Flowering time, plant growth and seed development
Corm weight	SNP 100026239	10	LSM6; U6 small nuclear RNA—associated sm-like protein	Contributes to yield indirectly by ensuring accurate RNA splicing and stable gene expression of pathways involved in growth development and stress responses
Corm weight	SNP 83978367	11	Zeb 1; Zinc finger E-box binding homeobox 1	Critical for efficient resource allocation and storage in plant
Total tuber weight	SNP 83978367			
Cormel weight	SNP 83979002	11	Cd2ap; CD2—associated protein	Involved in developing and maturing organs
Cormel weight	SNP 100202114	6	Dtx3l; deltex 3–like	Regulating protein levels and mediating cellular responses to various stimuli including development, immunity and stress responses such as nutrient stress and abiotic stresses

For number of suckers, the identified SNP marker on chromosome 3 harbors the General control non-depressible 1 (elF – 2 – alpha kinase activator) (Table 6).

For corm weight, the associated SNP marker on chromosome 10 harbors the sm-like protein U6 small nuclear RNA (LSM 6); on chromosome 11 harbors the zinc finger E-box binding homeobox 1 (Zeb1). Coincidentally, chromosome 11 also harbors zinc finger E-box binding homeobox 1 (Zeb1) for total tuber weight (Table 6).

For cormel weight, the identified SNP marker on chromosome 11 harbors the CD2-associated protein (Cd2ap) while the SNP markers on chromosome 6 harbors the Deltex 3-like (Dtx3l).

## Discussion

Taro leaf blight caused by the fungus–like oomycete *P. colocasiae*, has been reported as a major disease in taro^3,39^. To develop resistant cultivars, the QTL and genes associated with taro leaf blight resistance have to be identified. However, the study of taro genomics has been challenging due to the relatively large genomic size and high heterozygosity. With the development of long-read sequencing technologies and the high-throughput chromosome conformation capture (Hi-C) approaches, Yin et al.^40^ have successfully presented chromosome-level assembly of taro. Recently, GWAS peaks associated with yield per hectare, yield per plant, corm diameter, corm length, cormel diameter, cormel length, cormel weight, dry matter, number of cormels per plant, plant height, number of leaves per plant, number of suckers per plant, and petiole length were found in taro^29^.

In this study, there were seasonal effects on the performance and stability of taro genotypes. We started noticing the appearance of TLB disease on the leaves in early July. The first sign of the foliar disease was the presence of small dark lesions, which later increased in size and spread over the whole leaf during the peak of the rainy season. At the peak of the rainy season, precisely in August, the period of high humidity, the disease became very severe with milkish exudates at the back of the leaf where the lesions were. These observations are in agreement with Singh et al.^14^. The best 36 genotypes identified in the study, based on TLB resistance response and good establishment, were developed through crosses having Vanuatu and Samoa in their pedigree. These genotypes showed good resistance to the disease, although with differential responses. Three types of disease response were observed in the genotypes: (i) highly resistant genotypes, (ii) resistant genotypes, and (iii) moderately resistant genotypes. Both the highly resistant and resistant genotypes are generally low in disease symptoms, with the former almost symptomless during the peak of the disease pressure. The moderately resistant genotypes had a maximum score of 3 at the peak of disease pressure, but recovered after the rainy season, with the genotypes showing fewer disease symptoms. The disease response profile of the moderately resistant genotypes is an indication of a good and effective genetic mechanism in the disease response pattern.

Broad sense heritability measures the relative importance of nature versus nurture in the expression of a quantitative trait^41^. Estimates of broad-sense heritability for the taro leaf blight severity suggest a strong environmental influence and low genetic control. Marker-assisted selection could help to improve the selection response of this trait. A moderate broad-sense heritability was observed for vigor, indicating a good level of genetic control and potential for selection. A high heritability estimate was found in plant height, indicating that a large amount of the observable variance is due to genetic effects. The relatively low to moderate heritability observed for the yield traits (corm weight, cormel weight, and total tuber weight) and number of suckers reflects stronger environmental influences than genetic factors, highlighting the need for multi-environment evaluations to improve selection accuracy. Wide variability was exhibited in corm weight, cormel weight, and total tuber weight, indicating a rich pool of genetic diversity in the population.

The PCA biplot provided valuable insights into the interrelationships among traits and the genotypes in the taro population, and its interpretation was largely consistent with the pairwise correlation estimates. Yield traits such as cormel weight, corm weight, and total tuber weight clustered closely on the biplot and projected in the same direction along the first principal component, indicating strong positive associations. This was confirmed by the correlation matrix, where these traits showed significant positive correlations, suggesting that selection for one of these traits may result in improvements in the others. Taro leaf blight severity and yield traits were positioned in opposite quadrants, indicating negative associations with yield components, which were also evident in the correlation analysis. This is indicative that genotypes with high yield potential tend to be resistant/tolerant to TLB. According to Singh et al.^14^, TLB disease has the potential to create a devastating effect, such as a reduction in food production and household incomes, increased poverty, and even starvation in certain instances. Variance along the second principal component of the biplot was explained by vegetative traits like plant height and vigor, discriminating genotypes based on their growth characteristics. The significant negative correlation observed between TLB severity and vegetative traits such as vigor and plant height suggests that the resistant/tolerant genotypes were more vigorous and taller than the susceptible ones, whose growth and vigor were affected. Chiejine and Ugwuja^42^ revealed that individual taro accessions that had attained reasonable maturity and vigor before the outbreak of an infection would show more resistance than those in their juvenile stages. The biplot and correlation analysis highlight trait clusters that can be targeted simultaneously in selection, and provide genotypes that combine high yield potential, good morphological characteristics, and TLB resistance. The genotypes with resistance/tolerance to TLB attack would be used as parents in NRCRI taro breeding programmes to develop superior genotypes that exhibit good morphological traits, combine with TLB resistance, and high fresh tuber yield. This will support the Sustainable Development Goals (SDG) 2 by increasing food security and promoting sustainable agriculture, and SDG 3 by reducing health risks associated with chemical pesticides, promoting good health and well-being.

The SNPs summary statistics revealed important aspects of the genetic makeup and informativeness of the taro panel used in the study. The average minor allele frequency of 0.0575, with low mean polymorphic information content, indicates a high proportion of rare alleles in the population, leading to low-frequency variants and reduced recombination. Rare allele variants are essential for improving crop traits and uncovering the mechanisms underlying these traits^43^. Moderate values of observed and expected heterozygosity (Ho and He) indicate a measurable level of genetic variability in the population. In this study, we also found that the mean Ho has the same value as He, indicating that the population is in Hardy–Weinberg equilibrium. This means that the allele and genotype frequencies within the population are stable.

The population structure analysis is important to account for false positive associations between markers and traits in GWAS and serves as a guide for selecting genetically diverse parents in breeding programs. The population structure of the present study, based on the K clusters, revealed three clusters as the optimal genetic subgroups within the panel. The third cluster showed low admixture, suggesting a distinct ancestral background which were only derived from Samoa ancestry, while clusters 1 and 2 displayed shared ancestry that was likely caused by introgression among the genotypes as revealed by the pedigree information, which showed that the genotypes shared parents from different origins—Nigeria, Vanuatu, and Samoa. Similarly, the hierarchical clustering dendrogram analysis confirmed the evidence of subpopulation differentiation in the population. The observed clustering further suggests the influence of regional and geographic origin, local adaptation, and selection, consistent with earlier studies that reported genetic differentiation among taro germplasm from Asia and the Pacific^2,44^.

Recently, GWAS has become a central approach to studying the natural variation and mapping quantitative traits of root and tuber crops. In this study, a mixed linear model, population structure, and kinship matrices were used to control for false positives, thereby increasing the confidence in the significant associations. For TLB, there has been no prior report on the genomic regions associated with TLB resistance in any taro species; however, this study found 10 SNPs distributed across 8 chromosomes. Among these loci, six loci have been found to harbor genes such as Ferredoxin reductase (FDXR), Dihydrolipoamide dehydrogenase (DLD), Ornithine decarboxylase antizyme 2 (OAZ2), transient receptor potential (TRP) – like, Cathepsin B–like cysteine proteinase 5 and Ras oncogene family (RAB7B gene) that play essential role in keeping cell integrity, plant growth and development, responses to abiotic and biotic stresses and pathogen defense. In *Arabidopsis thaliana*, FDXR is required for proper chloroplast development and is involved in the regulation of plastid gene expression^45^. The DLD gene encodes an enzyme crucial for energy production and metabolic processes within the mitochondria. It also plays a role in the plant’s response to stress and defense mechanisms^46^. OAZ2 plays a part in regulating polyamines, which are crucial for the plant’s ability to adapt to and survive under stress conditions^47^. TRP is involved in several physiological conditions, such as cellular sensors, detecting environmental stimuli, and contributing to plant responses to stress, nutrient uptake, and cell signaling^48^. In the plant kingdom, plant development, germination, senescence, microspore embryogenesis, pathogen defense, and responses to abiotic stress, including programmed cell death, all involve cathepsin B^49^. Rab7 genes are involved in regulating cellular processes, including development and stress responses^50^. Studies in cotton (*Gossypium*) have shown that Rab7b genes play a role in drought tolerance by influencing the expression of stress–related transcription factors and the phytohormone abscisic acid^51^.

For the number of suckers, a significant SNP has been reported on chromosome 3 harboring a gene (General Control Non-derepressible 1 (GCN1) eIF–2–alpha kinase activator) that is essential for the plant’s response to nutrient deficiencies (nitrogen, phosphorus, and potassium) and other stresses^52^. This gene is also important for flowering, plant growth, and seed development in *Arabidopsis*^53^.

A total of seven significant SNPs were associated with yield traits, two SNP markers with corm weight, four SNP markers with cormel weight, and one SNP marker with total tuber weight. Of these seven loci, four SNP loci have been found to harbor genes (U6 small nuclear RNA-associated with sm-like protein (LSM6), Zinc finger E-box binding homeobox 1 (ZEB1), CD2-associated protein (CD2AP), and deltex 3–like (DTX3L)) that are crucial for plant growth and development, cell expansion and stress responses.

Sm-like protein specifically LSM6 was associated with corm weight. In *Arabidopsis*, this protein influences plant growth, biomass production, and yield^54,55^. SNP 83978367 was found to harbor the ZEB1 gene on chromosome 11 for corm weight. Coincidentally, the same gene was found on the same chromosome for total tuber weight. This co-localization of the SNP marker for corm weight and total tuber weight on chromosome 11 suggests either physical linkage or pleiotropic effects underlying their positive association. By modulating cell differentiation, ZEB1 can influence the formation of various tissues and organs that are critical for efficient resource allocation and storage in plants^56^.

For cormel weight, we found two SNP loci on chromosomes 6 and 11 (DTX3L and CD2AP), respectively. These genes are essential for organ development^57^, nutrient uptake, and abiotic stress responses. DTX3L, which functions as a ubiquitin ligase, plays a critical role in plant development and stress responses by regulating protein stability and activity^58^. CD2AP is widely expressed and differentially regulated during embryonic development^57^.

## Conclusion

This study is the first to apply a genome-wide association mapping approach to dissect the genetic basis of TLB resistance in taro. The genetic architecture of TLB resistance, number of suckers, corm weight, cormel weight, and total tuber weight is regulated by various SNPs distributed across the 14 chromosomes of the taro accessions evaluated in this study. GWAS led to the identification of eleven putative candidate genes associated with TLB resistance, number of suckers, corm weight, cormel weight, and total tuber weight in taro.

However, some limitations should be acknowledged. The phenotypic evaluations were conducted at a single location under natural field infestation across two years, which, while reflecting farmer-relevant conditions, may limit the extrapolation of the results to other diverse environments with possible different TLB pathogen strains. Yield-related traits exhibited low to moderate heritability, consistent with their polygenic nature and strong environmental influence, which may reduce the power to detect large-effect loci.

Despite these limitations, the identified marker–trait associations and candidate genomic regions provide a valuable foundation for future studies. Validation across multiple environments, controlled disease screening with different pathogen strains, and functional characterization of candidate genes will be essential to confirm the biological relevance of the associations and to facilitate their effective deployment in taro breeding programmes.

## Supplementary Information

Below is the link to the electronic supplementary material.

Supplementary Material 1

## Data Availability

The datasets generated and/or analysed during the current study are available in the European Variation Archive (EVA) repository with the project ID: PRJEB108862.
